# Effect of vitamin D on endothelial progenitor cells function

**DOI:** 10.1371/journal.pone.0178057

**Published:** 2017-05-17

**Authors:** Yoav Hammer, Alissa Soudry, Amos Levi, Yeela Talmor-Barkan, Dorit Leshem-Lev, Joel Singer, Ran Kornowski, Eli I. Lev

**Affiliations:** 1 "Sackler" Faculty of Medicine, Tel Aviv University, Ramat Aviv, Israel; 2 The Felsenstein Medical Research Institute, Petah-Tikva, Israel; 3 Cardiology institute, Rabin Medical Center, Beilinson/Hasharon Hospital, Petah-Tikva, Israel; 4 Endocrinology institute, Rabin Medical Center, Beilinson/Hasharon Hospital, Petah-Tikva, Israel; Centro Cardiologico Monzino, ITALY

## Abstract

**Background:**

Endothelial progenitor cells (EPCs) are a population of bone marrow-derived cells, which have an important role in the process of endothelialization and vascular repair following injury. Impairment of EPCs, which occurs in patients with diabetes, was shown to be related to endothelial dysfunction, coronary artery disease (CAD) and adverse clinical outcomes. Recent evidence has shown that calcitriol, the active hormone of vitamin D, has a favorable impact on the endothelium and cardiovascular system. There is limited data on the effect of vitamin D on EPCs function.

**Aim:**

To examine the in vitro effects of Calcitriol on EPCs from healthy subjects and patients with diabetes.

**Methods:**

Fifty-one patients with type 2 diabetes (60±11 years, 40% women, HbA1C: 9.1±0.8%) and 23 healthy volunteers were recruited. EPCs were isolated and cultured with and without calcitriol. The capacity of the cells to form colony-forming units (CFUs), their viability (measured by MTT assay), KLF-10 levels and angiogenic markers were evaluated after 1 week of culture.

**Results:**

In diabetic patients, EPC CFUs and cell viability were higher in EPCs exposed to calcitriol vs. EPCs not exposed to calcitriol [EPC CFUs: 1.25 (IQR 1.0–2.0) vs. 0.5 (IQR 0.5–1.9), p < 0.001; MTT:0.62 (IQR 0.44–0.93) vs. 0.52 (IQR 0.31–0.62), p = 0.001]. KLF-10 levels tended to be higher in EPCs exposed to vitamin D, with no differences in angiopoietic markers. In healthy subjects, calcitriol supplementation also resulted in higher cell viability [MTT: 0.23 (IQR 0.11–0.46) vs. 0.19 (0.09–0.39), p = 0.04], but without differences in CFU count or angiopoietic markers.

**Conclusion:**

In patients with diabetes mellitus, in vitro vitamin D supplementation improved EPCs capacity to form colonies and viability. Further studies regarding the mechanisms by which vitamin D exerts its effect are required.

## Introduction

Recent evidence indicates that circulating endothelial progenitor cells (EPCs), a population of bone marrow-derived cells, have an important role in the process of vascular repair, by promoting re-endothelialization following vascular injury [1]. EPCs are primarily identified by the expression of cell-surface antigenic markers, including CD133, CD34 and vascular endothelial growth factor receptor 2 (VEGFR-2), and have the ability to differentiate into mature cells with an endothelial phenotype [2]. Impairment of EPCs is related to endothelial dysfunction [3,4], coronary artery disease [5,6], heart failure [7] and adverse clinical outcome [8,9].

Findings from both experimental models and clinical studies support the hypothesis that the biology of EPCs is strongly related to the pathophysiology of coronary artery disease (CAD). Patients with CAD and CAD risk factors (smoking, diabetes, family history and hypertension) have significantly reduced levels of circulating EPCs compared with healthy individuals. Moreover, EPCs isolated from patients with CAD also revealed an impaired migratory response, which was inversely correlated with the number of risk factors [5]. Nevertheless, other studies have yielded conflicting results regarding the role of EPCs in CAD patients, indicating the need for further studies.

One possible mechanism by which EPCs is related to CAD is endothelial dysfunction. Endothelial dysfunction predicts cardiovascular (CV) events [10] and represents an underlying event for vascular abnormalities observed in cardiac and type 2 diabetes mellitus (DM) patients [11]. The circulating endothelial progenitor cells count has also been proposed as a surrogate marker of vascular dysfunction and is reduced in patients with various CV risk factors [3]. Furthermore, diabetes mellitus is known to have a deleterious effect on EPC function and level, and tight glycemic control was associated with an increase in EPCs levels and improvement in their functional properties [12].

Vitamin D is well known for its major role in bone mineral metabolism. However, in the last decade, it was demonstrated that vitamin D also confers additional effects, possibly dependent on the classical vitamin D receptors (VDRs) or by other and as yet not completely defined receptors expressed at the membrane level [13–15]. Moreover, recent studies have shown that both the synthesis of calcitriol (1,25(OH)2D) and the expression of VDRs are present in a large number of extra-renal sites (brain, prostate, colon, immune cells and endothelial cells [16]). Additional studies suggest a favorable impact of vitamin D on the CV system [17]. It was found that calcitriol may cause a regression of cardiac hypertrophy, thus reducing CV morbidity and mortality in patients with chronic renal failure who frequently suffer from accelerated atherosclerosis [17,18]. Furthermore, studies in human umbilical vein cord endothelial cells (HUVEC) suggested a significant effect of vitamin D on endothelial cells, in which Calcitriol was shown to have an anti-inflammatory effect in [19].

There is limited data on the effect of vitamin D on EPCs. We therefore aimed to investigate the effect of vitamin D on the functional aspects of EPCs, isolated from healthy subjects and from patients with diabetes mellitus, who are known to have attenuated EPC activity.

## Methods

### Patients

The study included 51 patients with diabetes mellitus (diabetes group) and 23 healthy volunteers (healthy group). In the diabetes group, only patients with treated type 2 diabetes mellitus (with insulin and/or oral hypoglycemic medications), 20–75 years of age with a baseline HbA1c level of ≥ 8% were eligible; while in the healthy group, healthy individuals without any background illnesses were included. Data regarding medical treatment was obtained from each patient, including prior oral vitamin D therapy. Exclusion criteria for the diabetes group were renal insufficiency (GFR <50 ml/min, calculated using the MDRD equation), hepatic dysfunction (alanine aminotransferase ≥ 2.5 times the upper limit of normal), thrombocytopenia (≤ 100×10^3^ cells/mm^3^), anemia (hemoglobin ≤ 10g/dl), recent acute coronary syndrome, coronary revascularization or stroke, and type 1 diabetes mellitus. Patients with malignant diseases or hematological disorders were also excluded. The study was approved by the Investigational Review Board and Ethics Committee of the Rabin Medical Center, and all subjects provided written informed consent.

### Blood sampling

Venous blood for EPC testing was drawn in heparinized tubes from an antecubital vein. All samples were processed within 1 hour of blood collection.

### EPC isolation

Peripheral mononuclear cells (PMNCs) were fractionated using Ficoll density-gradient centrifugation. Following red cell lysis, mononuclear cells were isolated and washed with phosphate-buffered saline. Isolated PMNCs were re-suspended with Medium 199 (Invitrogen, Carlsbad, California, USA) supplemented with 20% fetal calf serum (Gibco BRL Life Tech, Gaithersburg, Maryland, USA). Isolated cells were re-suspended in Medium 199 and plated on 6-well plates coated with human fibronectin at a concentration of 5 * 10^6^ cells per well.

### In vitro vitamin D supplementation

For each patient, 4 plates of EPCs were cultured; 2 plates were supplemented with supra-physiological (10^-9^mol/L) concentrations of calcitriol and 2 plates were not. All cultures were incubated for 7 days at 37^°^C with or without calcitriol supplementation.

### Colony forming unit quantification

Endothelial progenitor cell colonies were counted using an inverted microscope 7 days after plating. An EPC colony was defined as a cluster of at least 100 flat cells surrounding a cluster of rounded cells. A central cluster alone without surrounding cells was not counted as a colony. To confirm endothelial cell lineage, indirect immunostaining of randomly selected colonies was performed with antibodies directed against VEGFR-2, CD31 (Becton Dickinson, New Jersey, USA), and Tie-2 (Santa Cruz, Biotechnology, California, USA). Results were expressed as mean number of CFUs per well.

### MTT assay

The MTT assay was performed to evaluate viability of the cultured EPCs. MTT (3-[4,5-dimethylthiazol-2-yl]-2,5-diphenyl tetrazolium bromide) measures mitochondrial activity in living cells. After 7 days of culture, MTT (Sigma, St. Louis, Missouri, USA) 1 mg/mL was added to the EPC culture, and incubated for an additional 3–4 h. After incubation, the medium was removed and the cells solubilized in isopropanol; mitochondrial dehydrogenases of viable cells cleave the tetrazolium ring, yielding purple MTT crystals, which can be dissolved in isopropanol. The amount of dye released from the cells was measured with a spectrophotometer at 570 nm and subtracted background at 690 nm. An increase in the number of viable cells results in an increase in the amount of MTT formed and, therefore, in absorbance.

### Flow cytometry analysis for angiopoietic markers

After 7 days, the EPC culture was incubated with Tie-2 and VE-Cadherin antibodies, both angiopoeitic endothelial markers, for flow cytometry analysis. Flow cytometry analysis results were presented as the percentage of Tie-2/VE-Cadherin positive cells in EPCs supplemented with vitamin D, compared to EPCs not supplemented with vitamin D from the same patient.

### ELISA (enzyme-linked immunosorbent assay)

Further analysis was conducted on the effects of calcitriol on Kruppel-like factor 10 (KLF10); a transcription factor known to participate in various aspects of cellular growth, development, and differentiation. Previous studies regarding KLF10 and EPCs suggested it might play an important role in controlling EPCs differentiation and function [20]

### Statistical analysis

EPC parameters (flow cytometry determined levels, number of CFUs, and results of the functional assays) were not normally distributed, as determined by the Shapiro–Wilk normality test. Therefore, the EPC data are presented as median and interquartile range (IQR). Comparisons were performed using two-tailed Wilcoxon matched-pair signed rank tests (intra-group) or the Mann–Whitney–Wilcoxon tests (inter-group). Other parameters in the study and clinical variables were normally distributed, and therefore are presented as mean ± standard deviation (SD). All analyses were conducted using R: A language and environment for statistical computing, version 3.1.1 (R Foundation for Statistical Computing, Vienna, Austria) and p < 0.05 was considered statistically significant.

## Results

Fifty-one patients with diabetes mellitus and 23 healthy volunteers were recruited for the study during 2014–2016. Amongst patients with diabetes, the mean age was 60 ± 11 years, 60% were males, the mean HbA1C was 9.1 ± 0.8% and 21 patients (43%) were taking oral vitamin D (by drops, 400–800 IU per day) prior to their enrollment. Table 1 presents the baseline characteristics of the diabetes group. Mean age in the healthy group was 40 ± 11 years, and all participants were free of any medical history or current medical treatment.

**Table 1:** Baseline characteristics of study patients.

*Characteristic*	*Diabetic patients* *(n = 51)*
Age (years)	60 ± 11
Male Gender	31 (60%)
Ischemic heart Disease	16 (31%)
Hyperlipidemia	45 (89%)
Hypertension	33 (65%)
Mean eGFR (MDRD)*	96 ±21 ml/min
*Oral medical therapy*	
Aspirin	29 (57%)
Statin	45 (86%)
Ace inhibitor	15 (31%)
Angiotensin II receptor blocker	17 (34%)
Beta blockers	18 (36%)
Vitamin D	21 (43%)
Clopidogrel	2 (0.4%)
New oral anticoagulants	2 (0.4%)
*Anti-glycemic drugs*	
Metformin	36 (71%)
Insulin	36 (71%)
Sulfonylurea	18 (36%)
GLP-1 agonist	10 (21%)
DPP4 antagonist	13 (27%)

Values are mean±SD or n (%).

* eGFR, estimated glomerular filtration rate

MDRD; The Modification of Diet in Renal Disease equation.

**Fig 1:**
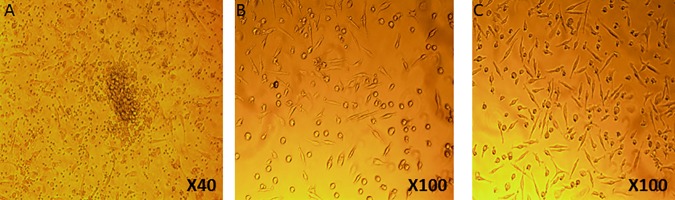
EPCs in light microscopy. EPCs in light microscopy after 7 days incubation in a healthy individual (A), a patient with diabetes mellitus (B) and in the same patient with diabetes after in vitro vitamin D supplementation (C).

Fig 1 depicts the morphological appearance of EPCs under light microscopy in a healthy volunteer and in a patient with diabetes mellitus, before and after in vitro vitamin D supplementation.

**Fig 2:**
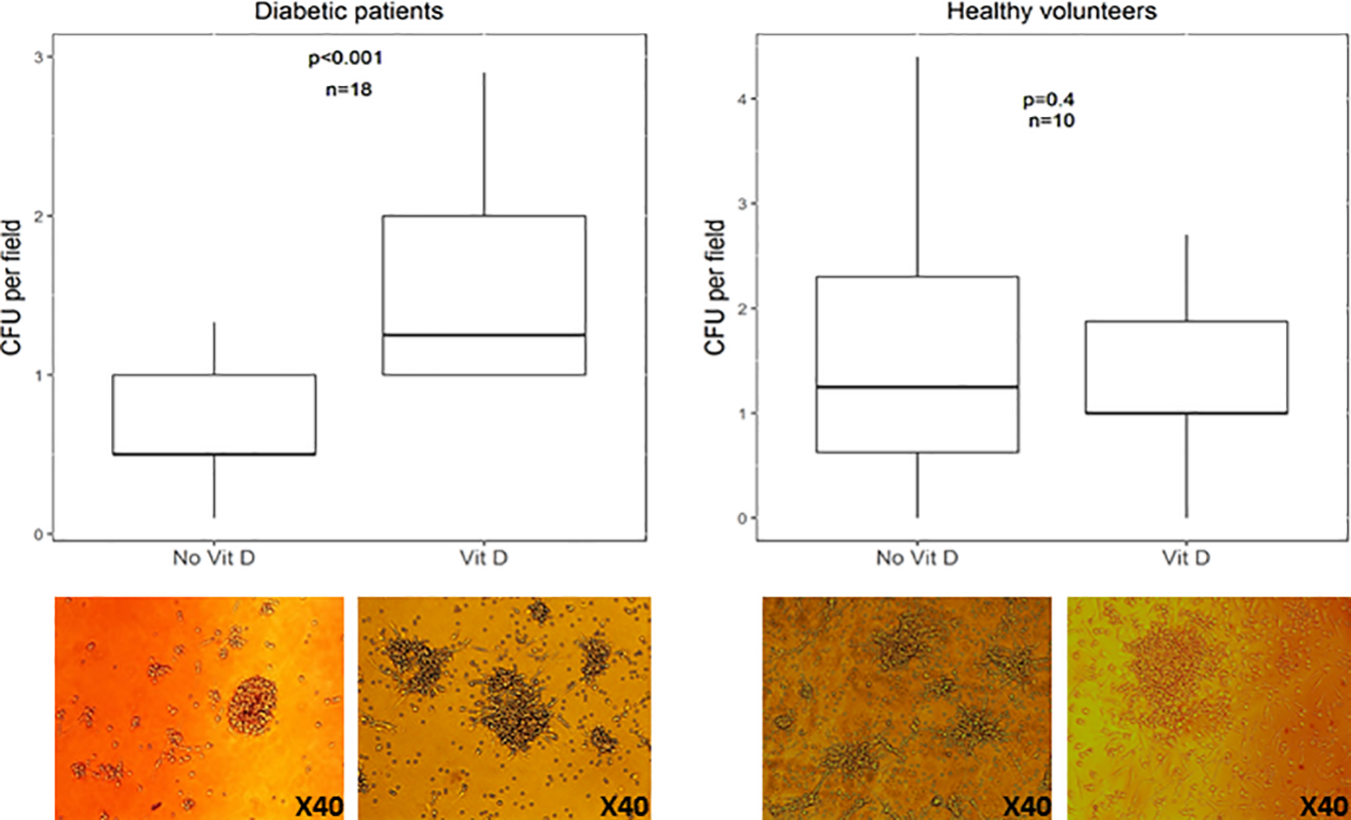
Colony forming units (CFU) in light microscopy. CFU per field as seen in light microscopy after 7 days incubation in patients with diabetes mellitus and healthy volunteers before and after in vitro vitamin D supplementation.

**Fig 3:**
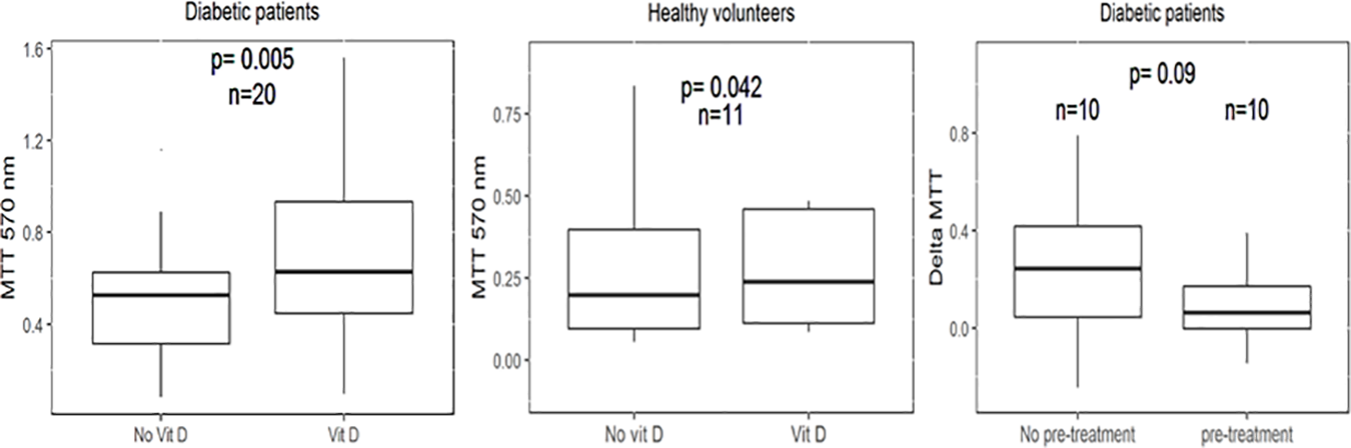
EPCs viability expressed by MTT assay. MTT in EPCs from patients with diabetes, with vs. without in vitro vitamin D supplementation (A). MTT in EPCs from Healthy volunteers, with vs. without in vitro vitamin D supplementation (B). The change in MTT values (delta MTT) after in vitro vitamin D supplementation in patients with diabetes mellitus who were previously treated with oral vitamin D vs those who were not (C).

In vitro exposure to vitamin D (calcitriol) was associated with higher CFUs and viability in EPCs for patients with diabetes [CFU count: 1.25 (IQR 1.0–2.0) vs. 0.5 (IQR 0.5–1.9), p value < 0.001, (Fig 2); MTT assay: 0.62 (IQR 0.44–0.93) vs. 0.52 (IQR 0.31–0.62), p = 0.001(Fig 3)]. While these measures improved significantly in patients with diabetes mellitus, the improvement in the healthy group was only noted in MTT values [0.23 (IQR 0.11–0.46) vs. 0.19, p = 0.042], with no significant difference in CFU count [1.25 (IQR 1.0–1.80) vs. 1.1 (IQR 0.60–2.30), p = 0.4].

The improved EPC viability (expressed by higher ΔMTT) was more pronounced (although without statistical significance) in patients not previously treated with oral vitamin D compared with patients already treated with oral vitamin D [MTT: 0.26 (0.03–0.44) vs. 0.04 (IQR 0.01–0.1), p = 0.09] (Fig 3).

**Fig 4:**
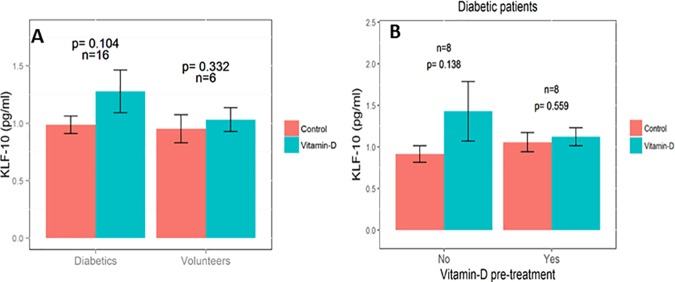
KLF-10 in EPCs. Enzyme-linked immunosorbent assay (ELISA) for kruppel like factor 10 (KLF-10) in EPCs with and without vitamin D supplementation in patients with diabetes Vs healthy volunteers (A) and the influence of oral vitamin D pre-treatment by the diabetes group on KLF-10 levels (B).

**Fig 5:**
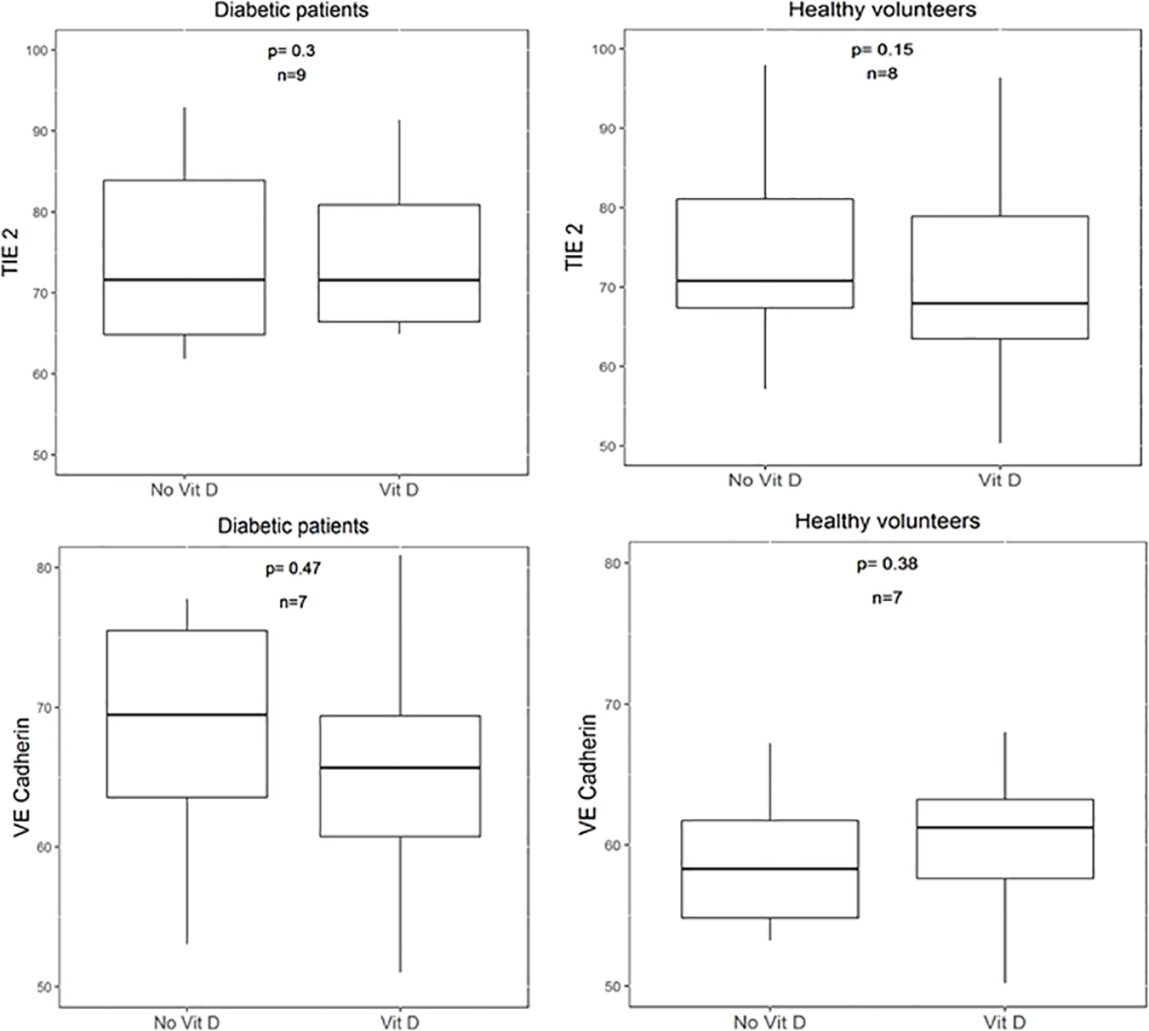
Angiopoietic markers in EPCs. Flow cytometry analysis for VE-Cadherin and TIE-2 in EPCs from patients with diabetes mellitus and healthy volunteers before and after in vitro vitamin D supplementation.

EPCs supplemented in vitro with vitamin D expressed numerically higher levels of KLF-10 in diabetic patients who were not treated with oral vitamin D prior to their enrollment. However, this difference did not reach statistical significance (Fig 4). We further performed a Flow cytometry analysis for TIE-2 and VE-Cadherin, which are angiopoietic markers exhibited by EPCs. However, there were no differences in these markers when comparing EPCs supplemented with vitamin D to those not supplemented in the diabetes group (Fig 5).

## Discussion

Little is known about the effect of vitamin D on the endothelial system, and specifically on EPCs. Our study shows that in vitro vitamin D supplementation improves functional parameters (such as colony forming capacity and viability) of EPCs in patients with diabetes mellitus and to a lesser extent in healthy subjects.

Previous studies displayed reduced levels of circulating EPCs with attenuated functional properties in patients with diabetes mellitus when compared to non-diabetics, and found that tight glycemic control improved their functional capabilities [12]. However, it is not known by which mechanisms diabetes mellitus affects these cells. Possible mechanisms suggested in previous studies are defective nitric oxide (NO) mediated EPC mobilization (from the bone marrow) and homing to injured vessels or tissues [21,22]; as well as increased apoptosis, impaired NO bioavailability, and reduced cell survival [23,24]. These impairments in EPC levels and functional properties may contribute to endothelial dysfunction, atherosclerotic disease progression, and attenuated wound healing observed in patients with diabetes.

Vitamin D has been thoroughly investigated regarding its beneficial cardiovascular effects, yet evidence is sparse regarding its influence on the endothelial system and EPC’s in particular. Previous studies that addressed this issue implied a possible role of vitamin D in EPC metabolism. Cianciolo et al. demonstrated an increased number of vitamin D receptors (VDR) on EPCs in hemodialysis patients treated with oral/IV vitamin D [25]; Yuen-Fung Yiu et al. demonstrated an inverse relationship between serum vitamin D level and circulating EPCs level in patients with diabetes mellitus [26]. Our study is the first to assess in vitro effects of vitamin D supplementation in patients with diabetes. In contrast to the positive effect on functional parameters of the cells, we did not observe any effects of calcitriol supplementation on EPC angiopoietic markers expression (VE-Cadherin, Tie-2). KLF-10 levels did not differ significantly between EPC's supplemented with in vitro vitamin D compared to those who were not, although EPCs from patients with diabetes who were not treated with oral vitamin D prior to their enrollment displayed a trend towards higher KLF-10 levels.

Several potential mechanisms may explain the effects of vitamin D on 37^o^ C. Firstly, as was suggested in our study, vitamin D may modulate KLF-10 levels, which in turn may stimulate activity of EPCs, an effect that was previously described as a possible stimulator of proangiogenic cells [20]. Secondly, vitamin D may modulate NO metabolism and blunt the effect of advanced glycosylation end products (AGE's) as was previously shown in human umbilical cord endothelial cells [19]. Another possible mechanism might relate to the role of vitamin D in modulation of endothelial proinflammatory transcription factor nuclear factor κB (NFkB) as was previously shown in the study of Jablonski et al. [27].

Regardless of the exact mechanism, the relation between in vitro vitamin D supplementation and the improvement of EPCs functional properties may suggest the pathway by which vitamin D exerts its beneficial effects on the cardiovascular system, possibly by attenuating endothelial dysfunction. The fact that in vitro vitamin D supplementation in our study did not improve angiopoietic cellular markers expression on EPCs (Tie-2, VE-Cadherin) invites further studies regarding the mechanisms by which vitamin D influences EPCs and endothelial cells. The pathway of KLF10 may be modulated by vitamin D, although our study failed to establish this relation with certainty. Further studies are also required to examine the effect of in vivo vitamin D treatment on EPC's and the endothelial system.

Our study has several limitations. First, we lack data regarding serum vitamin D levels at time of enrollment in the "diabetes group" and in the "healthy volunteers group". Second, this study assessed only the in vitro effects of vitamin D on EPCs.

## Conclusion

In vitro vitamin D supplementation improved EPCs viability and ability to form colonies in patients with type 2 diabetes mellitus, and to a lesser extent in healthy subjects. Further studies regarding the mechanisms by which vitamin D exerts its effect are required.

This research received no specific grant from any funding agency in the public, commercial, or not for-profit sectors.

## Supporting information

S1 File Minimal dataset diabetics. (XLSX)

S2 File Minimal dataset healthy. (XLSX)
